# Persistent astrocytic IL-3 stimulation of microglia slows disease in Alzheimer’s: treatment perspectives for Alzheimer’s

**DOI:** 10.1038/s41392-021-00806-x

**Published:** 2021-11-09

**Authors:** Natja Haag, Hans Zempel

**Affiliations:** 1 Institute of Human Genetics, Medical Faculty, RWTH Aachen University, Aachen, Germany; 2 Institute of Human Genetics, Faculty of Medicine and University Hospital Cologne, University of Cologne, Cologne, Germany; 3 Center for Molecular Medicine Cologne (CMMC), Faculty of Medicine and University Hospital Cologne, University of Cologne, Cologne, Germany

**Keywords:** Diseases of the nervous system, Neurological disorders, Medical genetics

**Fig. 1:**
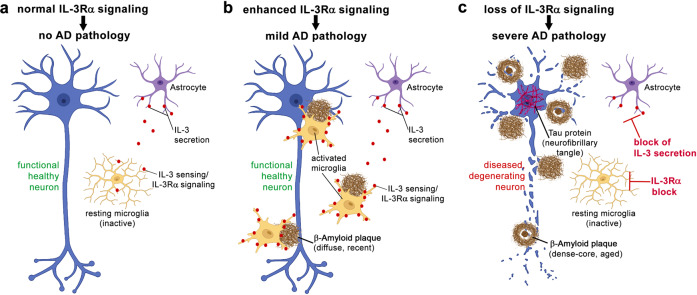
Blocking of either astrocytic IL-3 secretion or microglial IL-3 sensing by IL-3Rα results in worsened AD pathology in disease paradigms. **a** In conditions without typical AD pathology or stimuli, microglia are not triggered to become active. **b** In AD conditions, permissive IL-3/IL-3Rα signaling allows microglia to be transcriptionally reprogrammed and activated, which results in enhanced microglial motility and clustering of microglia around plaques, and as a likely consequence relatively mild AD pathology/plaque load. **c** Unphysiological, complete block of either astrocytic IL-3 secretion or microglial IL-3 sensing by IL-3Rα both results in microglia that do not adequately respond to the presence of AD pathology, microglia remain quiescent, likely resulting in relatively severe AD pathology/plaque load

In their new paper, McAlpine and colleagues provide compelling evidence that astrocyte dependent microglial stimulation is crucial for fighting off one of the most prominent Alzheimer disease (AD) pathological hallmarks, namely β-amyloid (Aβ) plaque deposition. They find that (i) a subpopulation of astrocytes secrete the classical cytokine interleukin-3 (IL-3), (ii) microglia are responsive to this cytokine, (iii) responsiveness (not so much secretion of IL-3) is increased in patients with AD and model systems of AD, and (iv) presence of IL-3 is necessary to allow microglia to counteract some of the detrimental effects of AD: if IL-3 is missing, AD pathology worsens (see Fig. 1 for graphical depiction).^1^

AD and related tauopathies, e.g., frontotemporal dementia (FTD) or frontotemporal lobar degeneration with tauopathy (FTLD-TAU), are detrimental diseases, impose a huge burden on patients and caregivers, and are the scourge of modern healthcare systems. Despite recent advances in (immuno-) therapies, a causative cure seems out of range. For AD, a major event in disease progression is the deposition of extracellular plaques mainly composed of the fibrillogenic peptide Aβ, cleaved out of the amyloid-precursor-protein (APP). The microtubule-associated-protein TAU, an intracellular axonal protein with a pleiotropy of functions, i.a. the modulation of microtubule dynamics, mislocalizes into the soma (so-called TAU-missorting), becomes phosphorylated, and eventually forms intracellular neurofibrillary tangles (NFTs). NFTs and TAU-missorting are associated with neuronal dysfunction, loss of synapses, and neurodegeneration.^2^ Strong genetic evidence both from the human disease and mouse models put Aβ/APP-processing and TAU in the center of disease causes: all genetic forms of AD are due to mutations in the *APP-*gene or genes involved in the processing of APP (*PSEN1, PSEN2*), while mutations in *MAPT*, the gene coding for the TAU protein, are sufficient to cause related dementia syndromes, i.e., FTD variants like Progressive Supranuclear Palsy or variants of FTLD-TAU. TAU is a crucial mediator of Aβ/APP-processing pathology: TAU-KO models are resistant against Aβ-induced neuronal dysfunction. It appears thus reasonable that many therapeutic endeavors today focus on Aβ/APP-processing and TAU.

The huge majority of cases of AD and related dementia syndromes are, however, not genetic in terms of typical monogenetic inheritance patterns. Yet, heritable factors could be responsible for the bigger part of an individual’s risk for AD,^3^ and polygenic risk calculations are gaining traction. In the last decades, identified risk genes shifted part of the attention of the field to potentially disease modifying pathways. While some risk genes are clearly related to APP and TAU processing, proper microglial function has emerged as potentially crucial for preserving brain function also in disease context. Among the genes important for microglial functions, *TREM2* is one of the most important ones, but recent data suggests that of the 75 genes possibly involved in modifying AD risk, many are related to microglial function.^4^ Microglia are the resident macrophages of the brain, and are critically involved not only in the context of pathogen defense, but also in basic brain homeostasis and the formation, development, and pruning of synapses. Both detrimental and beneficial action of microglia in dementia have been described.

What is IL-3? In the periphery, IL-3 is mainly produced by activated T- and B-cells, is capable, i.a., of stimulating the proliferation of a multitude of immune cell types, inducing various effector functions to adapt defense against microbial pathogens, and aiding platelet reconstitution via induction of megakaryocytes. Like other signaling molecules, IL-3 too, has a different function in the brain. Starting from the 80ies, when IL-3 was shown to be produced by astrocytes, the IL-3 cytokine was implicated in AD and could in some studies even hold off neurodegeneration and critical TAU pathology.^5^

How does IL-3 act? IL-3 (signaling) acts downstream of Trem2: McAlpine et al. showed that in response to increasing Aβ plaque deposition, Trem2 signaling increases microglial IL-3 responsiveness via upregulation of the IL-3 receptor alpha (IL-3Rα).^1^ The subsequent functional changes of microglia result in immune regulation via transcriptional reprogramming and, i.a., increased motility and migration towards Aβ plaques.^1^ Genetic suppression of either astrocytic secretion of IL-3, or microglial expression of IL-3Rα or Trem2 results in decreased clustering of microglia around Aβ-plaques, increased plaque load and worsened cognition in animals.^1^ With this novel evidence from the study by McAlpine et al., it can now be safely assumed that IL-3 presence and Trem2-mediated increases in IL-3Rα signaling is crucial to fight off some of the detrimental effects of Aβ plaque deposition. The study also clearly shows that brain IL-3 is not of peripheral origin but secreted by a subpopulation of astrocytes and, as also shown by the discussed paper, secretion increases with age, but is unchanged in AD conditions or models. Brain IL-3 does not respond to peripheral inflammation.^1^

While this excellent paper clarifies the role of IL-3 and in part also of microglia in the AD pathogenesis, unfortunately, IL-3 itself does not appear to be of direct therapeutic value. IL-3 does not readily cross the blood brain barrier, and increasing the IL-3 brain presence via intrathecal delivery of recombinant IL-3 only appears to be beneficial when endogenous IL-3 had been ablated.^1^ Also, this study uses a model of AD (the so-called 5xFAD-mouse-model, harboring 3 pathogenic mutations in *APP* and 2 pathogenic mutations in *PSEN1*, but no mutation in *MAPT*), which does not faithfully recapitulate the typical TAU pathology crucial for human disease. The study is thus focused on Aβ pathology. What can we learn from this study? It is tempting to speculate that e.g. in mild or early stage of human disease, increasing the suggested signaling pathway via other means, e.g. pharmacologically, would result in disease modification. It remains unclear (i) whether the brain has already exploited to the maximal extend its physiological mechanisms to maintain (microglia-based) brain homeostasis and cognitive function (as IL-3 sensitivity is already increased in disease), (ii) how to pharmacologically or otherwise activate microglia just to the right extend, and (iii) how to activate just the right microglial functions to counteract disease. For a therapeutic approach, recruiting disease-countering functions of microglia (without triggering detrimental brain inflammation) appears currently more challenging than targeting upstream events in disease, namely (i) the early oligomerization and aggregation of the Aβ-peptide, and (ii) the early mislocalization and late aggregation of the neuronal-dysfunction mediating protein TAU.
